# Aortic Valve Replacement vs. Balloon-Expandable and Self-Expandable Transcatheter Implantation in Low-Risk Patients

**DOI:** 10.3390/jcm14238278

**Published:** 2025-11-21

**Authors:** Vittoria Lodo, Enrico Giuseppe Italiano, Luca Weltert, Edoardo Zingarelli, Cristina Viscido, Gabriella Buono, Paolo Centofanti

**Affiliations:** 1 Department of Cardiac Surgery, Mauriziano Hospital, 10128 Turin, Italy; edzingarelli@hotmail.com (E.Z.); centofantipaolo@gmail.com (P.C.); 2 Division of Cardiac Surgery, Department of Cardiac, Thoracic, Vascular Sciences and Public Health, University of Padova, 35122 Padua, Italy; italiano.enrico.1993@gmail.com; 3 Department of Cardiovascular Sciences, European Hospital, 00142 Rome, Italy; lweltert@gmail.com; 4 Department of Cardiovascular Anesthesia and Intensive Care, Mauriziano Hospital, 10128 Turin, Italy; cristina.viscido@gmail.com (C.V.); gbuono@mauriziano.it (G.B.)

**Keywords:** aortic valve stenosis, low-risk, SAVR, balloon-expandable TAVI, self-expandable TAVI

## Abstract

**Objectives:** This study sought to compare midterm outcomes of low-risk patients who underwent a surgical aortic valve replacement (SAVR) vs. balloon-expandable (BE) or self-expandable (SE) transcatheter aortic valve implantation (TAVI). **Methods:** Data on consecutive patients undergoing SAVR or transfemoral TAVI between 2017 and 2022 were collected. Patients were separated into three groups according to the type of prosthesis: a biological surgical prosthesis, BE prosthesis and SE prosthesis. The three groups were compared in terms of baseline characteristics, post-procedural outcomes and long-term survival. **Results:** A total of 542 patients were enrolled, and 221 received a surgical prothesis, 150 received a BE prosthesis and 171 received an SE prosthesis. TAVI patients were older and had a higher risk profile compared to surgical patients. Propensity score matching resulted in an excellent matching of nearly 80 patients in each group. In the matched cohort, SE prostheses were associated with a significantly higher incidence of stroke (SE group 6.3%, BE group 0, SAVR group 2.3%, *p* = 0.045), para-valvular leak (SE group 8.1%, BE group 2.4%, SAVR group 0, *p* = 0.017) and left bundle branch block (SE group 23.8%, BE group 18.2%, SAVR group 0%, *p* < 0.001). Regarding 5-year mortality, no significant differences were reported between the BE and SE TAVI (13.6% vs. 22.5%, *p* = 0.066). However, when comparing surgery versus TAVI, the SE prosthesis showed a significantly higher 5-year mortality (22.5% vs. 11.6%, *p* = 0.042). Instead, the BE prosthesis demonstrated its non-inferiority compared to the surgical prosthesis (13.6% vs. 11.6%, *p* = 0.249). **Conclusions:** The BE prosthesis should be considered the prosthesis of choice for patients with a long life expectancy requiring a transcatheter procedure.

## 1. Introduction

Over the last decade, advancements in transcatheter aortic valve implantation (TAVI) have led to an expansion of its indication, now including patients with severe aortic stenosis (AS) who are at low surgical risk, with encouraging results in this population [1,2].

The extension of the TAVI indication to low-risk patients is associated with an increase in the number of procedures performed in patients who are younger and have a longer life expectancy compared to those who have received first-generation prostheses. Consequently, there is a pressing need to optimize both procedural planning and prostheses’ design to minimize the incidence of peri-procedural complications, such as para-valvular leak (PVL) and conduction abnormalities, which are related to worse long-term outcomes.

Nevertheless, even if the incidence of these complications appears to have decreased in the latest randomized clinical trials (RCTs) [1,2], enrolling low-risk patients and using last-generation devices, such untoward issues still generate some concerns for the extension of TAVI indications to patients with a longer life expectancy.

Two main types of transcatheter heart valves (THVs) are commercially available: balloon-expandable valves (BEVs) and self-expandable valves (SEVs). BEVs rely on the radial force provided by the accompanying balloon to achieve expansion. In contrast, SEVs deploy automatically and continue to expand until they meet resistance from the annular wall, allowing them to conform to the anatomical features of the aortic wall [3].

Whether these two very different THV concepts are achieving similar or different clinical outcomes remains unclear, and controversial data are reported in the literature [4,5,6].

Furthermore, while most of the available studies report only early and short-term outcomes of transcatheter procedures, there is a growing interest in long-term outcomes with the extension of TAVI indications to low-risk patients.

On the other hand, the long-term durability of bioprosthetic surgical valves has been proved in several studies [7,8].

The present study sought to compare the post-procedural outcomes and midterm survival of low-risk patients who underwent an SAVR or TAVI using BE or SE prostheses.

## 2. Method

### 2.1. Patient Population and Study Design

From September 2017 to December 2022, data on consecutive patients undergoing SAVR or transfemoral (TF) TAVI for severe AS were prospectively collected and retrospectively analyzed.

Patients were eligible for inclusion in this study if they had an isolated severe AS; a low surgical risk, defined as Euroscore II or STS score < 4 [9,10]; and were aged between 70 and 85 years. Exclusion criteria were the need for concomitant surgical or transcatheter procedures, a history of cardiac surgery, valve-in-valve procedures and a requirement of urgent or emergent surgery.

Patients were divided into 3 groups according to the type of prosthesis they received: surgical bioprosthesis, BE TAVI or SE TAVI. The 3 groups were compared in terms of pre-operative characteristics, early post-operative outcomes and midterm survival.

Each patient was allocated to the most appropriate approach after an accurate multidisciplinary evaluation based on clinical history, blood tests, electrocardiogram, echocardiogram, computed tomography (CT) and cardiac catheterization.

This study was conducted according to the guidelines of the Declaration of Helsinki and approved by the local Ethic Committee at the Mauriziano Hospital, Turin, Italy (Protocol number 260-2022).

All patients received a follow-up visit at 3 and 6 months and an annual telephone survey. The follow-up was completed in March 2025.

### 2.2. Operative Technique

All patients underwent surgery or TF TAVI according to our standardized approach [11,12].

Briefly, all TAVIs were performed using TF approach under conscious sedation. Both new-generation BE (Sapien, Edwards Lifesciences, Irvine, CA, USA) and SE (Evolut [Medtronic, Minneapolis, MN, USA], Portico/Navitor [Abbott, Chicago, IL, USA]) prostheses were implanted. Balloon aortic valvuloplasty, before and/or after TAVI, was performed at operator discretion. The choice of prosthesis type and size was mainly based on CT scan.

SAVR was performed through a mid-sternotomy or full sternotomy. Both bovine pericardial valves, such as Carpentier–Edwards Magna Ease (Edwards Lifesciences, Irvine, CA, USA) and Avalus (Medtronic, Minneapolis, MN, USA), and porcine valves, such Epic bioprosthesis (Abbott, Chicago, IL, USA), have been implanted. The following 2 suture techniques were used: a continuous suture technique by means of 3 sutures of 0 polypropylene or an interrupted suture by means of single stiches of 2/0 non-absorbable polyester suture with ventricular side pledgets.

### 2.3. Endpoints and Definitions

The primary endpoint of this study was midterm all-cause mortality. Secondary endpoints were post-procedural complications such as acute kidney injury (AKI), stroke, myocardial infarction (MI), permanent pacemaker (PPM) implantation, new-onset left bundle branch block (LBBB) and para-valvular leak (PVL) greater than mild. AKI was defined according to Kidney Disease: Improving Global Outcomes (KDIGO) criteria [13].

Peri-operative MI was recorded in case of cTn-T value > 10 times the 99th percentile of the upper reference limit during the first 48 h with ECG abnormalities and/or angiographic or imaging evidence of new myocardial ischemia or/and new cases of myocardial viability [14].

### 2.4. Statistical Analysis

For continuous variables, data were presented as mean and interquartile range (IQR), and for categorical variables, data were reported as counts and percentage. Differences between groups were assessed using Student’s *t*-test and Chi-square test or Fisher’s exact test, as appropriate.

To balance the distribution of baseline risk factors between groups, propensity score (PS) matching was performed. PS matching was performed by running a logistic binary regression with the prosthesis type as dependent variable; the probability of the regression was stored and used as matching score for the best neighbor matching. The overall efficacy of the match method was then tested by re-running the logistic regression and verifying that no variables had a significant difference. The a priori selected variables were age, gender, hypertension, chronic kidney disease (CKD), Euroscore II and STS score.

Midterm survival function was assessed and reported using the Kaplan–Meier method, and the survival curves were compared using the log-rank test (Mantel–Cox).

A *p* value of <0.05 was considered statically significant.

The statical analysis was performed using SPSS Version 27.0 (IBM Corp., Armonk, NY, USA).

## 3. Results

### 3.1. Overall Population

During the study period, 542 patients with a diagnosis of severe isolated AS underwent an SAVR (*n* = 221, 40.8%), BE TAVI (*n* = 150, 27.7%) or SE TAVI (*n* = 171, 31.5%).

The baseline characteristics and comorbidities are reported in Supplemental Material Table S1.

Both SE and BE TAVI patients were significantly older compared to surgical patients (SE group 81 [80–84]; BE group 81 [78–83]; SAVR group 75 [71–78], *p* < 0.001). Patients who received an SE prosthesis were more commonly female (SE group 65.5%; BE group 37.3%; SAVR group 45.2%, *p* < 0.001) and had a higher incidence of hypertension (SE group 94.7%, BE group 86%, SAVR group 90.5%, *p* = 0.028) when compared to patients who received a BE TAVI or a surgical bioprosthesis.

Patients who underwent SE or BE TAVI had a higher Euroscore II (SE group 2.22 [1.74–3.01]; BE group 2.16 [1.62–2.90]; SAVR group 1.77 [1.27–2.43], *p* < 0.001) and a higher STS score (SE group 2.26 [1.87–3.07]; BE group 2.34 [1.75–3.1]; SAVR group 1.58 [1.1–2.54], *p* < 0.001).

Regarding post-operative outcomes, surgical patients had a higher incidence of post-procedural AKI compared to BE and SE TAVI patients (SAVR group 16.7%; BE group 2%; SE group 4.1%, *p* < 0.001). SE TAVIs were associated with a significantly higher incidence of stroke (SE group 5.8%; BE group 0.7%; SAVR 2.7%, *p* = 0.026) and a PVL ≥ 2 (SE group 9%; BE group 2.8%; SAVR 0.5%, *p* < 0.001) compared to the BE TAVI and surgical bioprosthesis. Patients who received an SE or BE prosthesis were more likely to develop an LBBB (SE group 24%; BE group 22%; SAVR group 0.9%, *p* < 0.001) and to require PPM implantation (SE group 12.3%, BE group 9.3%; SAVR group 3.6%, *p* = 0.005) compared to surgical patients.

The post-operative outcomes are reported in Supplemental Material Table S2.

### 3.2. Matched Population

After propensity score matching, 85 surgical patients were compared to 88 patients with a BE TAVI and 80 patients who received an SE TAVI.

The baseline characteristics of the matched population are reported in Table 1.

The propensity score matching resulted in a good matching of all pre-procedural variables, including for important prognostic factors such as age, cardiovascular risk factors, kidney function, Euroscore II and STS scores.

The post-procedural outcomes of the matched population are reported in Table 2.

Surgical patients still had a significantly higher incidence of post-procedural AKI (SAVR group 23.3%, BE group 2.3%, SE group 5%, *p* < 0.001). However, no significant differences were reported in terms of the need for RRT between the three groups.

SE prostheses were associated with a significantly higher incidence of post-procedural stroke (SE group 6.3%, BE group 0, SAVR group 2.3%, *p* = 0.045) and PVLs (SE group 8.1%, BE group 2.4%, SAVR group 0, *p* = 0.017).

BE prostheses were still associated with a higher risk of post-procedural LBBBs compared to surgery; however, its rate remained significantly lower than in the SE TAVI (BE group 18.2%, SE group 23.8%, SAVR group 0%, *p* < 0.001).

Regarding midterm survival, no significant differences were reported between the SE and BE TAVI (77.5% vs. 86.4%, *p* = 0.546).

**Figure 1:**
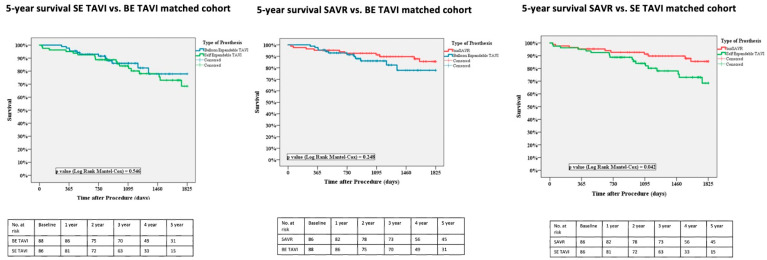
Kaplan–Meier midterm survival curves for SAVR vs. BE TAVI and SE TAVI patients, matched cohort. SAVR: surgical aortic valve replacement, TAVI: transcatheter aortic valve implantation, BE: balloon-expandable and SE: self-expandable.

When comparing surgery with TAVI, SE prostheses were associated with a significantly shorter midterm survival than surgical bioprostheses (77.5% vs. 88.2%, *p* = 0.042). Instead, BE prostheses demonstrated their non-inferiority in terms of midterm survival compared to surgery (86.4% vs. 88.2%, *p* = 0.248) (Figure 1).

## 4. Discussion

The present study comprises a retrospective single-center PS-matched comparison of the post-procedural outcomes and midterm survival of low-risk patients with severe AS who underwent an SAVR, BE TAVI or SE TAVI.

The main findings of this study are as follows: (i) BE prostheses are shown to be non-inferior to surgery in regard to all-cause midterm mortality, whereas SE prostheses are associated with a significantly higher midterm mortality compared to the SAVR; (ii) a PVL is more common after TAVI compared to surgery, with SE TAVI being associated with a significantly higher incidence of PVLs when compared to BE prostheses; (iii) patients who received an SE TAVI are at a higher risk of post-procedural stroke; (iv) the risk of a post-procedural LBBB is higher for all TAVI prostheses compared to surgery but is also significantly higher for SE prostheses compared to BE prostheses.

The encouraging results of recent RCTs [1,2] comparing TAVI and SAVR in low-risk patients have accelerated the expansion of TAVI indications to include younger patients. As a result, the latest ESC/EACTS guidelines provide a Class I recommendation for TAVI in patients over 70 years or those with a high surgical risk [15].

Despite the growing enthusiasm for transcatheter approaches, the broad use of TAVI in younger and low-risk patients remains controversial. TAVI has been linked to higher rates of new PM implantation, LBBB, PVL and stroke. While such risks may be considered acceptable in patients at prohibitive, high and intermediate surgical risk, their impact in patients with a longer life expectancy is still a source of concern.

Furthermore, while the long-term durability of bioprosthetic surgical valves and the satisfactory life expectancy after SAVR have been confirmed in several studies [16,17], for TAVI, the long-term durability beyond 5–8 years is poorly understood [18,19].

Recently, Thyregod and collogues published the 10-year results of the NOTION trial [20]. They found that in low-risk patients, the rate of major clinical outcomes was similar between SAVR and TAVI after 10 years. However, the incidence of severe structural valve deterioration was lower with TAVI. Despite these findings, the NOTION trial has several limitations. Like other RCTs, it included a highly selective patient population that does not reflect real-world scenarios. Moreover, it involved surgical bioprostheses such a Trifecta and Mitroflow, both known for their poor long-term durability [21,22]. Additionally, only a limited number of patients completed the 10-year follow-up. These limitations weaken the comparison between TAVI and surgery, and the NOTION trial data are insufficient to support the use of TAVI in patients with long life expectancies.

In this scenario, identifying the type of THV that can offer the optimal long-term outcomes and represents a valuable alternative to surgery is becoming increasingly important.

Our results regarding the higher rate of PVLs in SE TAVI, likely due to their lower radial force, are in line with the literature. The lower radial force of SE TAVI probably reduces the prosthesis’s effectiveness in overcoming valve calcification and may explain the higher incidence of post-procedural PVLs.

In the CHOICE trial, the rate of more than mild aortic regurgitation was 18% in SE prostheses and 4% in BE prostheses [23].

In their meta-analysis, Sà and collogues compared last-generation BE and SE valves. BE TAVIs were associated with a significantly higher incidence of PVLs when compared to SE prostheses (4.9% vs. 11.3%, *p* < 0.001) [24].

Lerman et al. performed a meta-analysis comparing short- and midterm outcomes of BE and SE TAVI; 21 publications totaling 35.248 patients were included. The rate of moderate–severe PVLs was significantly higher in patients with SE prostheses when compared to those with a BE TAVI (8.1% vs. 4.4%, *p* < 0.001) [25].

The SOLVE TAVI is a multicentric randomized trial of 447 patients undergoing TF TAVI, comparing SE and BE valves. It reports similar results in terms of moderate–severe PVL distributions between the two study groups (SE group 3.4%; BE group 1.5%; *p* = 0.001) [26].

Van Belle and colleagues compared the outcomes of BE and SE valves using a large nationwide registry [6]. In the PS-matched cohort, they found a significantly higher risk of PVLs with SE TAVI when compared to BE TAVI, irrespective of valve generation.

PVLs have been strongly related to poorer short- and long-term outcomes [27,28].

The sudden onset of a PVL following TAVI in patients with pre-existing severe AS, whose left ventricles are not adapted to volume overload, may explain the significant negative effect on PVLs in this group of patients. While chronic aortic regurgitation can remain asymptomatic for years, even with left ventricle enlargement and dysfunction, acute aortic regurgitation due to a PVL in a left ventricle that is not “preconditioned” can quickly lead to decompensated heart failure and adverse outcomes.

Furthermore, in our analysis, LBBBs occur more frequently in SE TAVI patients when compared to BE TAVI patients. Our results are in line with the literature and are related to the different implantation techniques, with a greater implantation depth in SE TAVI causing a higher risk of injury to the conduction system [29,30]. In their study, Bendandi and colleagues aimed to establish a new score to predict the occurrence of conduction abnormalities after TAVI, and they identified SE TAVI as an independent predictor [31].

It has been widely demonstrated that patients with new-onset persistent LBBBs after TAVI have a greater risk of all-cause mortality, as well as a greater incidence of cardiovascular mortality, heart failure hospitalization and PM implantation [32].

The higher rate of such complications in patients with SE prostheses, together with the higher incidence of post-procedural stroke, may explain their higher midterm mortality rates compared to surgery. On the other hand, the lower rate of post-procedural PVLs, LBBBs and stroke in BE valves compared to SE TAVI should explain their non-inferiority in terms of midterm survival when compared to surgery.

Our results are corroborated by several studies reported in the literature.

In the meta-analysis by Lerman and colleagues, SE TAVIs were associated with a significantly higher short term all-cause mortality rate when compared to BE TAVI (3.4% vs. 2.4%, *p* < 0.001) [25].

In their study, including 31,113 patients, Deharo et al. found that BE valves were associated with lower rates of all-cause mortality, cardiovascular death and re-hospitalization for heart failure compared with SE valves [33].

Similar results were reported in a meta-analysis by Jacquermyn et al. that included 26 studies. BE prostheses showed a better overall survival and a lower cardiovascular death rate when compared to SE prostheses [34].

Furthermore, in their propensity score-matched analysis, Van Belle et al. demonstrated that the use of SE valves was associated with a significantly higher in-hospital and 2-year mortality rate compared to BE valves [6].

Finally, when approaching TAVI in younger lower risk patients, it is important to keep in mind that these patients are at a high risk of requiring subsequent coronary angiography and percutaneous coronary interventions.

Several retrospective and single-center studies have reported difficulties in coronary access after TAVI with tall-frame compared to short-frame TAVI [35,36].

These results have been recently confirmed by the CAvEAT registry [37], which demonstrated a clear advantage in the coronary re-access of BE TAVI, with its intra-annular, short-frame design minimizing interference with coronary ostia.

The primary limitation of our study is its single-center, retrospective and observational design and the absence of randomization. Although propensity score matching was applied to ensure comparable and balanced risk profiles between the three groups, the influence of unmeasured confounding variables on the outcomes cannot be entirely ruled out. Another limitation is the small sample size, which is due to this study’s single-center design. Furthermore, the midterm follow-up period is limited, and a longer follow-up period is mandatory to better understand long-term outcomes in low-risk patients with longer life expectancies.

## 5. Conclusions

Despite technical advancements and device innovation, conduction abnormalities and PVLs remain the TAVI’s Achille’s hell.

With the extension of TAVI indications to patients with long life expectancies, it has become crucial to identify the type of transcatheter prosthesis that can offer the best long-term outcomes and serve as a valid alternative to surgery.

Based on our analysis, BE prostheses should be considered the prostheses of choice for patients with long life expectancies requiring a transcatheter procedure. Indeed, BE valves showed a lower rate of peri-procedural complications when compared to SE valves and demonstrated their non-inferiority in terms of midterm survival when compared to surgery. Obviously, further studies with a longer follow-up period are needed before routinely recommending BE TAVI for this category of patients.

## Figures and Tables

**Table 1:** Baseline characteristics and comorbidities.

Variables	SAVR (*n* = 85)	BE TAVI (*n* = 88)	SE TAVI (*n* = 80)	*p* Value
Age, median (IQR), years	78 (76–80)	79 (77–81)	79 (78–81)	0.062
Female, *n* (%)	40 (46.5)	35 (39.8)	41 (51.2)	0.323
BMI, mean (SD), kg/m^2^	26.4 (3.3)	26.5 (4.1)	26.4 (2.8)	0.675
Hypertension, *n* (%)	79 (91.9)	81 (92.0)	78 (97.5)	0.240
Diabetes, *n* (%)	24 (27.9)	26 (29.4)	23 (28.7)	1.000
Dyslipidemia, *n* (%)	42 (48.8)	50 (56.8)	39 (48.8)	0.477
Smoke, *n* (%)	27 (31.4)	29 (32.9)	28 (35)	0.849
COPD, *n* (%)	10 (11.6)	16 (18.2)	16 (20.0)	0.306
PAD, *n* (%)	15 (17.4)	21 (23.9)	14 (17.5)	0.475
CKD (eGFR < 60 mL/min), *n* (%)	24 (27.9)	17 (19.3)	26 (32.5)	0.142
RRT, *n* (%)	1 (1.2)	0	1 (1.2)	0.497
History of cerebrovascular event, *n* (%)	8 (9.3)	10 (11.4)	8 (10)	0.975
History of coronary disease, *n* (%)	9 (10.5)	12 (13.63)	11 (13.7)	0.915
RBBB, *n* (%)	6 (6.9)	7 (7.9)	8 (10)	0.767
Mean gradient, mean (IQR), mmHg	48 (44–52)	46 (42–50)	46 (41–48)	0.647
AVA, mean (IQR), cm^2^	0.70 (0.38–0.93)	0.68 (0.33–0.95)	0.70 (0.41–0.95)	0.723
LF-LG, *n* (%)	5 (5.8)	7 (7.9)	5 (6.2)	0.998
NYHA III-IV, *n* (%)	38 (44.2)	33 (37.5)	29 (36.3)	0.524
History of heart failure, *n* (%)	12 (14.0)	15 (17.0)	16 (20.0)	0.583
EF, median (IQR)	60 (56–65)	60 (58–65)	60 (55–65)	0.715
Euroscore II, median (IQR)	2.21 (1.56–3.06)	1.94 (1.51–2.55)	2.17 (1.63–2.80)	0.337
STS score, median (IQR)	2.19 (1.65–2.78)	1.89 (1.49–2.68)	2.15 (1.67–2.89)	0.356

SAVR: surgical aortic valve replacement, TAVI: transcatheter aortic valve indication, BE: balloon-expandable, SE: self-expandable, IQR: interquartile range, BMI: body mass index, SD: standard deviation, COPD: chronic obstructive disease, PAD: peripheral artery disease, CKD: chronic kidney disease, eGFR: estimated glomerular filtration rate, RRT: renal replacement therapy, RBBB: right bundle branch block, AVA: anatomic valvular area, LF-LG: low flow–low gradient, NYHA: New York Heart Association and EF: ejection fraction.

**Table 2:** Post-operative outcomes.

Variables	SAVR (*n* = 85)	BE TAVI (*n* = 88)	SE TAVI (*n* = 80)	*p* Value
AKI, *n* (%)	20 (23.3)	2 (2.3)	4 (5.0)	<0.001
RRT	2 (2.3)	0	1 (1.2)	0.089
Stroke, *n* (%)	2 (2.3)	0	5 (6.3)	0.045
MI, *n* (%)	2 (2.3)	2 (2.3)	2 (2.5)	0.896
New onset LBBB, *n* (%)	0	16 (18.2)	19 (23.8)	<0.001
PM implantation, *n* (%)	4 (4.7)	11 (12.5)	10 (12.5)	0.139
PVL (≥mild to moderate), *n* (%)	0	2 (2.4)	6 (8.1)	0.017

SAVR: surgical aortic valve replacement, TAVI: transcatheter aortic valve indication, BE: balloon-expandable, SE: self-expandable, AKI: acute kidney injury, RRT: renal replacement therapy, MI: myocardial infarction, LBBB: left bundle brunch block, PM: pace-maker and PVL: para-valvular leak.

## Data Availability

The data that support the findings of this study are available from the corresponding author upon reasonable request.

## Supplementary Materials

The following supporting information can be downloaded at https://www.mdpi.com/article/10.3390/jcm14238278/s1: Table S1: Overall population baseline characteristics and comorbidities. Table S2: Overall population post-procedural outcomes.
