# Supplementary material for: Aortic Valve Replacement vs. Balloon-Expandable and Self-Expandable Transcatheter Implantation in Low-Risk Patients
Source: J Clin Med. 2025 Nov 21;14(23):8278. doi: 10.3390/jcm14238278 (PMC12693321; doi:10.3390/jcm14238278)
Supplement: Supplementary file 1 [file jcm-14-08278-s001.zip › jcm-3965013-supplementary.pdf]

Table S1: baseline characteristics and comorbidities

Variables	SAVR (n=221)	BE TAVI (n=150)	SE TAVI (n=171)	p value
**Age, median (IQR), years**	75 (72-78)	81 (78-83)	81 (80-84)	<0.001
**Female, n (%)**	100 (45.2)	56 (37.3)	112 (65.5)	<0.001
**BMI, mean (SD), kg/m²**	26.5 (3.4)	26.4 (4.3)	26.3 (5.2)	0.565
**Hypertension, n (%)**	200 (90.5)	129 (86.0)	162 (94.7)	0.028
**Diabetes, n (%)**	45 (20.4%)	38 (25.3%)	37 (21.6)	0.175
**Dyslipidemia, n (%)**	107 (48.4)	83 (55.3)	84 (49.1)	0.384
**Smoke, n (%)**	48 (21.7%)	35 (23.3%)	37 (21.6)	0.376
**COPD, n (%)**	35 (15.8)	24 (16.0)	25 (14.6)	0.928
**PAD, n (%)**	37 (16.7)	31 (20.7)	26 (15.2)	0.415
**CKD (eGFR<60 ml/min) , n (%)**	38 (17.2)	45 (30.0)	64 (37.4)	<0.001
**RRT, n (%)**	4 (1.8)	3 (2.0)	4 (2.33)	0.867
**History of cerebrovascular event, n (%)**	20 (9.0)	16 (10.6)	18 (10.5)	0.845
**History of coronary disease, n (%)**	31 (14.0%)	18 (12)	23 (13.4)	0.998
**RBBB, n (%)**	20 (9.3%)	17 (11.3)	18 (10.5)	0.778
**Mean gradient, mean (IQR), mmHg**	48 (42-53)	46 (41-53)	47 (43-52)	0.637
**AVA, mean (IQR), cm²**	0.69 (0.33-0.97)	0.71 (0.31-0.95)	0.73 (0.31-0.91)	0.788
**LF-LG, n (%)**	15 (6.8%)	10 (6.7)	11 (6.4)	0.997
**NYHA III-IV, n (%)**	89 (40.3)	51 (34.0)	64 (37.4)	0.472
**History of heart failure, n (%)**	28 (12.7)	23 (15.3)	27 (15.8)	0.634
**EF, median (IQR)**	60 (56-65)	60 (58-65)	60 (59-65)	0.351
**Euroscore II,median (IQR)**	1.77 (1.27-2.43)	2.16 (1.62-2.90)	2.22 (1.74-3.01)	<0.001
**STS score, median (IQR)**	1.58 (1.1-2,54)	2.34 (1.75-3.1)	2.26 (1.87-3.07)	<0.001

Table S1. SAVR: surgical aortic valve replacement; TAVI: transcatheter aortic valve indication, BE: balloon-expandable; SE: self-expandable; IQR: interquartile range, BMI: body mass index, SD: standard deviation; COPD: chronic obstructive disease, PAD: peripheral artery disease, CKD: chronic kidney disease, eGFR: estimated glomerular filtration rate; RRT: renal replacement therapy, RBBB: right bundle branch block, AVA: anatomic valvular area, LF-LG: low flow-low gradient, NYHA: New York Hear Association, EF: ejection fraction

Table S2: post-operative outcomes

Variables	SAVR (n=221)	BE TAVI (n=150)	SE TAVI (n=171)	p value
**AKI,n (%)**	37 (16.7)	3 (2.0)	7 (4.1)	<0.001
**Stroke, n (%)**	6 (2.7)	1 (0.7)	10 (5.8)	0.026
**MI, n (%)**	4 (1.8)	4 (2.7)	5 (2.9)	0.750
**New onset LBBB, n (%)**	2 (0.9)	33 (22.0)	41 (24.0)	<0.001
**PM implantation, n (%)**	8 (3.6)	14 (9.3)	21 (12.3)	0.005
**PVL (≥ mild to moderate), n %)**	1 (0.5)	4 (2.8)	15 (9.0)	<0,001

Table S2. SAVR: surgical aortic valve replacement; TAVI: transcatheter aortic valve indication, BE: balloon-expandable; SE: self-expandable; AKI: acute kidney injury; MI: myocardial infarction, LBBB: left bundle brunch block, PM: pacemaker, PVL: para-valvular leak
